# Comparison of Matrix Frequency-Doubling Technology (FDT) Perimetry with the SWEDISH Interactive Thresholding Algorithm (SITA) Standard Automated Perimetry (SAP) in Mild Glaucoma

**Published:** 2017

**Authors:** Azadeh DOOZANDEH, Farnoosh IRANDOOST, Ali MIRZAJANI, Shahin YAZDANI, Mohammad PAKRAVAN, Hamed ESFANDIARI

**Affiliations:** 1 Ophthalmology Department, Shahid Beheshti University of Medical Sciences, Tehran, Iran; 2 Optometry Department, Shahid Beheshti University of Medical Sciences, Tehran, Iran; 3 Optometry Department, Iran University Of Medical Sciences, Tehran, Iran; 4 Ophthalmic Epidemiology Research Center, Shahid Beheshti University of Medical Sciences, Tehran, Iran

**Keywords:** Mild Glaucoma, Matrix-FDT, Visual Field, SITA-SAP, SWEDISH Interactive Thresholding Algorithm

## Abstract

This study aimed to compare second-generation frequency-doubling technology (FDT) perimetry with standard automated perimetry (SAP) in mild glaucoma. Forty-seven eyes of 47 participants who had mild visual field defect by SAP were included in this study. All participants were examined using SITA 24-2 (SITA-SAP) and matrix 24-2 (Matrix-FDT). The correlations of global indices and the number of defects on pattern deviation (PD) plots were determined. Agreement between two sets regarding the stage of visual field damage was assessed. Pearson’s correlation, intra-cluster comparison, paired t-test, and 95% limit of agreement were calculated. Although there was no significant difference between global indices, the agreement between the two devices regarding the global indices was weak (the limit of agreement for mean deviation was -6.08 to 6.08 and that for pattern standard deviation was -4.42 to 3.42). The agreement between SITA-SAP and Matrix-FDT regarding the Glaucoma Hemifield Test (GHT) and the number of defective points in each quadrant and staging of the visual field damage was also weak. Because the correlation between SITA-SAP and Matrix-FDT regarding global indices, GHT, number of defective points, and stage of the visual field damage in mild glaucoma is weak, Matrix-FDT cannot be used interchangeably with SITA-SAP in the early stages of glaucoma.

## INTRODUCTION

Glaucoma is a chronic life-long optic neuropathy because degeneration of ganglion cells and optic nerve impairment occurs slowly [1, 2]. People become aware of their problem after involvement of the central visual field [3]. Visual field testing by using a technique named perimetry helps locate the damaged areas [4]. Many patients with glaucoma may have to undergo various visual field tests during follow-up. Standard automated perimetry (SAP) is widely applied to diagnose and follow-up glaucoma. The most commonly acquired perimetric test is SAP with the Swedish interactive thresholding algorithm (SITA) strategy [5]. In the 1990s, frequency-doubling technology (FDT) perimetry was introduced to detect field loss due to glaucoma earlier than SAP [6]. The conceptual basis of FDT is the frequency-doubling illusion that is due to presentation of a sinusoidal grating with a low spatial frequency at a high temporal frequency. This type of presentation produces a perceived image that appears double its actual spatial frequency [7]. One function of the magnocellular pathway is the ability to perceive this illusion. However, this ability is lost in the early stages of glaucoma [8]. Large stimulus size, low spatial resolution, and reduced potential to localize the defects have limited the clinical profitableness of first-generation FDT [9, 10]. With the second version of FDT, Matrix FDT-2, each target subtends 5 degrees of visual angle instead of the 15 degrees in the previous version and the stimuli are presented as a pattern as in SAP [11]. Several studies have compared FDT with SAP [12, 13]. Some compared full-threshold automated perimetry [12], but this technique is not commonly used in today’s glaucoma practice. Compared to full-threshold automated perimetry, the SITA strategy has shorter test duration and higher reliability and clinical accuracy [14]. Some other studies compared the N-30 version of FDT in which each target subtends a large area of about 15 degrees [13]. In this study, visual field test results obtained through Matrix-FDT were compared with those obtained through SITA-SAP in mild primary glaucoma. The question behind this study was whether smaller targets distributed on Matrix-FDT 24-2 in a pattern similar to that of the SITA SAP 24-2 test would produce any visual field defect comparable with that obtained by SITA SAP 24-2 until clinicians would be able to establish a relationship between the results of these tests during follow-up.

## MATERIALS AND METHODS

Forty-seven eyes of 47 patients with primary open-angle glaucoma (POAG) in the mild stage based on Hodapp–Parrish–Anderson classification [15] were included in this cross-sectional study. The study protocol was approved by the Ethics Committee of the Ophthalmic Research Center of Shahid Beheshti University of Medical Sciences and written consent was obtained from each patient. All measurements were obtained in one single day, and all participants were enrolled within a 6-month period from November 2013 until April 2014 in Labbafinejad Medical Center, Shahid Beheshti Medical University, Tehran, Iran. All participants had undergone at least one SAP before the study so they were familiar with the procedure. None of the participants had experience with Matrix-FDT testing before the study. A brief display was made for the participants to get them familiarized with the procedure. The participants who met the inclusion criteria and who agreed to participate in the study were enrolled.

To have a power of 90% to detect a 1-unit difference in mean deviation (MD) between two sets when the standard deviation of the difference was assumed to be 2.1, a sample size of 47 was calculated.

The inclusion criteria were as follows: best-corrected visual acuity (BCVA) of logMAR 0.3 or better, spherical refraction within 3 diopters (D), and cylinder correction within 3 D. A glaucoma specialist examined all participants, including VA assessment, slit-lamp biomicroscopy, intraocular pressure (IOP) measurement with Goldmann applanation tonometry, gonioscopy with the Zeiss four-mirror glass, and dilated fundus examination 1 day before the tests. Reliable visual fields (fixation losses, false-positives, and false-negatives all less than 15%) were included. Hodapp–Parrish–Anderson criteria [15] were used to determine the severity of visual field loss on SAP. Participants with mild visual field defects on SAP were included (MD <-6 dB, fewer than 25% of pattern deviation (PD) points under the 5% level and less than seven points under the 1% level, no points with sensitivity <15 in the central 5°). Participants with other intraocular abnormalities, a disease that might affect visual function testing (e.g., pituitary lesions, ischemic optic neuropathy, diabetic retinopathy), and low test reliability on previous visual fields were excluded from the study.

The visual field of all participants was assessed by Matrix-FDT (Humphrey Matrix, Carl Zeiss Meditec, and Jena, Germany) and the Humphrey Field Analyzer II (Carl Zeiss Meditec) in random order. To remove the effect of fatigue during the tests, a 10-minute break was allowed between each test. The selection of each eye was performed randomly. Stimuli of SITA-SAP were Goldmann size III (0.43°) on a 31.5-apostilb background, which consists of 52 test locations. Stimuli of FDT perimetry were 0.25 cyc/deg grating with phase flickers at 25 H. FDT perimetry was performed using the 24-2 full-threshold program with 5° stimuli at 55 test locations. The matrix- FDT (24-2) uses an algorithm for estimation of threshold. The blind spot and central visual field point are two locations that are tested by FDT perimetry, but not by SAP.

The global indices of visual field and Glaucoma Hemifield Test (GHT) measured by matrix-FDT and SAP test were compared. The magnitude of the visual field defect between FDT and SAP and the number of defects at P < 0.5, P < 0.2, P < 0.1, and P < 0.05 in each quadrant was summed up and compared. FDT visual fields were assessed based on the Brusini classification [16]. Stage of VF defect detected by FDT was compared with that of SAP. Sensitivity threshold in every point of the visual field in the FDT was compared with the corresponding point in SAP.

The agreement between SITA-SAP and Matrix-FDT was evaluated in patients with POAG with early visual field defect by statistical analysis. To present data, mean and standard deviation were used. To correlate the agreement of two devices regarding global indices, hemifields, quadrants, sectors, and single field points, we used Pearson’s correlation, intracluster correlation (ICC), paired t-test, 95% confidence interval of difference, and limit of agreement (LoA). SPSS (Version 21.0, IBM Co., Chicago, IL) was used for all statistical analysis. A P-value less than 0.05 was regarded as statistically significant.

## RESULTS

**Figure 1:**
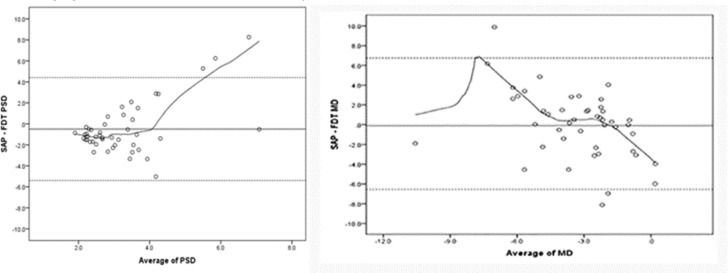
A: Bland–Altman Plot of Pattern Standard Deviation (PSD) in Matrix Frequency-Doubling Perimetry (FDT) versus Swedish Interactive Thresholding Algorithm (SITA) Standard Automated Perimetry (SAP).

Forty-seven eyes of 47 POAG participants with mild visual field damage confirmed by SAP were included in this study. There were 19 males and 28 females. The mean age of the participants was 53.1 ± 16.2 years. The spherical equivalent of refractive error was -0.21 ± 1.88 and BCVA (log MAR) was 0.14 ± 0.08. Although there was no significant difference between global indices, the agreement between the two devices regarding global indices was weak (Table 1 and Fig 1).

**Figure 2:**
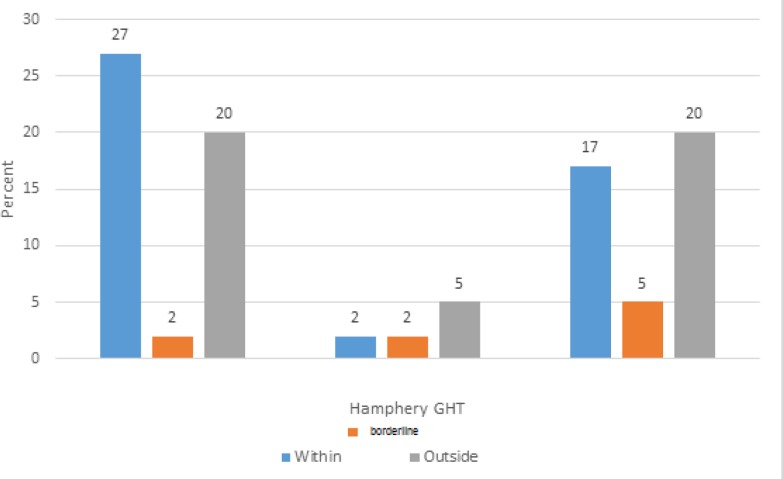
Agreement between the Matrix Frequency-Doubling Perimetry (FDT) versus Swedish Interactive Thresholding Algorithm (SITA) Standard Automated Perimetry (SAP) Regarding the Glaucoma Hemifield Test (GHT).

The LoA for MD was -6.08 to 6.08 and that for pattern standard deviation (PSD) was -4.42 to 3.42. The Bland–Altman plot shows that there was no agreement between SITA-SAP and Matrix-FDT regarding MD and PSD. In addition, this discrepancy had a trend with increasing MD and PSD values (Fig 1A and 1B). The agreement between SITA-SAP and Matrix-FDT regarding GHT is shown in Table 2 and Fig 2.

**Figure 3:**
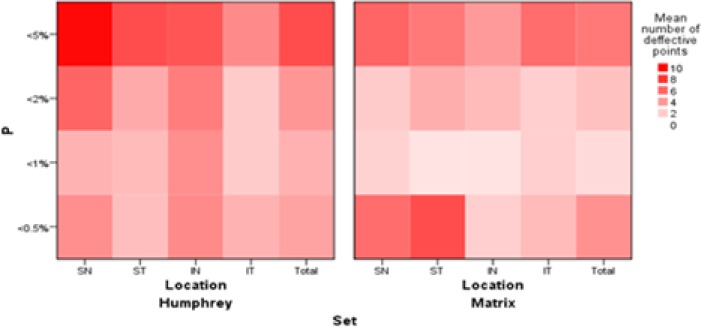
The Number of Defective Points with P < 5%, P < 2%, P < 1%, and P < 0.5% at the Superionasal (SN), Superiotemporal (ST), Inferionasal (IN), and Inferiotemporal (IT) Quadrants by the Matrix Frequency-Doubling Perimetry (FDT) versus Swedish Interactive Thresholding Algorithm (SITA) Standard Automated Perimetry (SAP).

Only 48.8% of the participants had the same results of GHT on the two sets (kappa = 0.121). The number of defective points at P < 5%, P < 2%, P < 1%, and P < 0.5% in supratemporal (ST), supranasal (SN), infratemporal (IT), and infranasal (IN) quadrants is shown in Fig 3.

**Figure 4:**
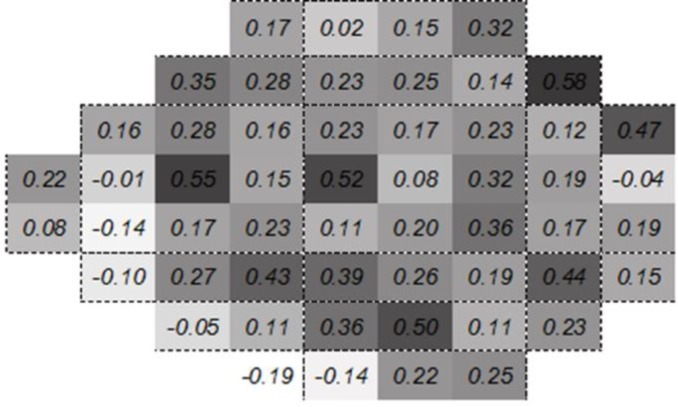
Pointwise Correlation of Matrix Frequency-Doubling Perimetry (FDT) versus Swedish Interactive Thresholding Algorithm (SITA) Standard Automated Perimetry (SAP) in measurement of Mean Deviation (MD) within Different Locations of the Visual Field.

The correlation between the two devices in terms of visual field defects was clinically weak (partial correlation = 0.322, P < 0.001) in most of the points (only in 7 points out of 54 an r > 0.4 and P < 0.05 was observed). Point-wise comparison of corresponding locations obtained by two sets was weak in most of the points, indicating the non-agreement of the measurement of sensitivity of the points by the two sets (Fig 4).

Among the 47 study participants with confirmed early VF defect on SITA-SAP, Matrix-FDT visual field defects were classified as normal in 13 (27%), early damage in 22 (46%), moderate damage in 9 (19%), and severe damage in 3 (6%).

**Table 1:** Global Indices and Test Duration of SITA-SAP and Matrix-FDT

	Mean ± SD	Median	Minimum	Maximum	P*
MD					
SITA-SAP	-3.31 ± 2.1	-3.01	-11.52	0.11	0.905
Matrix-FDT	-3.37 ± 3.25	-3.31	-11.96	3.18	
PSD					
SITA-SAP	3 ± 2.13	2.12	1.08	10.92	0.169
Matrix-FDT	3.51 ± 1.12	3.12	2.33	7.34	
Test duration (s)					
SITA-SAP	347.3 ± 53.7	341	259	482	0.001
Matrix-FDT	319.9 ± 21.3	314	294	387	

MD: mean deviation, PSD: pattern standard deviation, S: seconds, Matrix-FDT: matrix frequency doubling perimetry, SITA: Swedish interactive thresholding algorithm, SAP: standard automated perimetry

B: Bland–Altman Plot of Mean Deviation (MD) in Matrix-FDT versus SITA-SAP.

**Table 2:** Agreement of MD and PSD between Matrix Frequency-Doubling Perimetry (FDT) and Swedish Interactive Thresholding Algorithm (SITA) Standard Automated Perimetry (SAP)

**Parameter of interest**	**Correlation of two sets**	∆ ** Mean ± SD**	**∆ Range**	**Absolute ∆ Mean ± SD**	**P-value** *	**95% CI**	**ICC**	**LoA (lower to upper)**
**MD**	0.259	0.1 ± 3.4	-8.1 to 9.9	2.5 ± 2.2	0.905	-0.94 to 1.06	0.06	-6.56 to 6.76
**PSD**	0.071	-0.5 ± 2.5	-5 to 8.3	1.9 ± 1.6	0.169	-1.24 to 0.22	0.24	-5.4 to 4.4

∆ : Difference between SITA-SAP and Matrix-FDT, LoA: limit of agreement, MD: mean deviation, PSD: pattern standard deviation, SD: standard deviation, CI: confidence interval,

* Based on the paired t-test

Within: within normal limit, Outside: outside normal limit

## DISCUSSION

In this study, despite no significant difference between the global indices of the visual fields, the correlations between the two sets were weak in global indices (MD and PSD), GHT, number of defective points, point-wise comparison of corresponding points, and staging of the VF damage. Matrix-FDT has high sensitivity and specificity compared to SITA-SAP to detect visual field deficits [11]. Because Matrix-FDT (30-2 screening protocol) evaluates 19 locations while SITA-SAP (30-2 protocol) evaluates 76, direct comparison of Matrix-FDT findings with SITA-SAP findings was difficult. By introduction of a newer generation of FDT, which uses a smaller target similar to that of SITA-SAP, Matrix-FDT could be used with the benefit of detecting mild glaucomatous VF abnormalities. However, a correlation between Matrix-FDT and SITA-SAP was not detected in our study. Matrix-FDT and SITA-SAP measure different entities of retinal sensitivity. Frequency-doubling technology uses contrast sensitivity threshold to counter phase flickering of sinusoidal waves [7], while SITA-SAP uses white light intensity. Therefore, the threshold values obtained by each device were not expected to be directly comparable, as was shown by point-wise comparison of the thresholds in the fields. The two tests measure the severity of glaucomatous damage of the visual field. Hence, some similarity between the results of the two sets was expected. The number of defects on the pattern deviation plots was assessed because this map has already been set according to each machine’s normative database. Nevertheless, the correlation between defects was not found in the current study. One might attribute this discrepancy to the ability of the FDT to detect VF deficit prior to SAP. Several studies suggested that in the early stages of glaucoma, Matrix-FDT detected the locations of selective loss of retinal ganglion cells in the magnocellular pathway more extensively in comparison to the defects of VF loss detected by SAP [17, 18]. Our results cannot support this theory. In the current study, 27% of the fields, which were normal with FDT had glaucomatous VF defect on SAP.

Our results are not in agreement with findings of Artes et al. that say there is no systematic difference between FDT and SAP with regard to PD [19]. However, our results are in line with those of Leeprechanon et al. and Patel et al. Leeprechanon et al. [11] reported that in the glaucoma group, the defect score on the pattern deviation plot was higher on FDT than on SAP. However, Patel et al. [20] reported that 30% of abnormal SITA fields could not be detected by Matrix-FDT. Case selection may account for the similarity between FDT and SAP in some studies. In a study by Lester et al. [21], in which normal or ocular hypertension participants were included, by definition no VF defect should be visible on the visual fields. Therefore, whenever no defect is visible on the field, the results of the VF testing by different instruments should be very similar. One possible explanation for the poor correlation between the two sets in this study might be inexperience of the participants with Matrix-FDT. Our study participants were experienced with SITA-SAP, but not with FDT. Favorable variability characteristics with small learning effects have been reported for first-generation FDT in participants with perimetric experience [22]. However, test practice sessions might decrease the variability of the test results and improve the correlation between the two sets. Another limitation of our study is that we did not perform conformational visual field testing. High test variability was reported for the SAP, which despite a reliable visual field could result in different field defect [19, 22]. Our study included participants with mild visual field damage on SAP (that was our limitation), which enabled us to evaluate the agreement of two sets in fairly uniform participants. With glaucoma progression, test-retest variability would increase, and worse agreement between the two sets would be expected. Advantages of FDT are its portability and quick procedure, which increases patient acceptance [23]. Furthermore, it has a role in the early diagnosis of glaucoma. However, our study shows that FDT cannot be used interchangeably with SAP in the early stage of glaucoma because the correlation between the two sets is weak in any parameter of the VF.

In conclusion the correlation between Matrix-FDT and SITA-SAP in terms of global indices, GHT, number of defective points, and stage determination of the visual field damage is weak. Therefore, Matrix-FDT cannot be used interchangeably with SITA-SAP in the mild stage of glaucoma during follow-up.
